# Supplementary figures and images for: Google searches for suicide and suicide risk factors in the early stages of the COVID-19 pandemic
Source: PLoS One. 2020 Jul 24;15(7):e0236777. doi: 10.1371/journal.pone.0236777 (PMC7380602; doi:10.1371/journal.pone.0236777)

Supplement 1: Weekly observed and predicted relative Google search proportions.


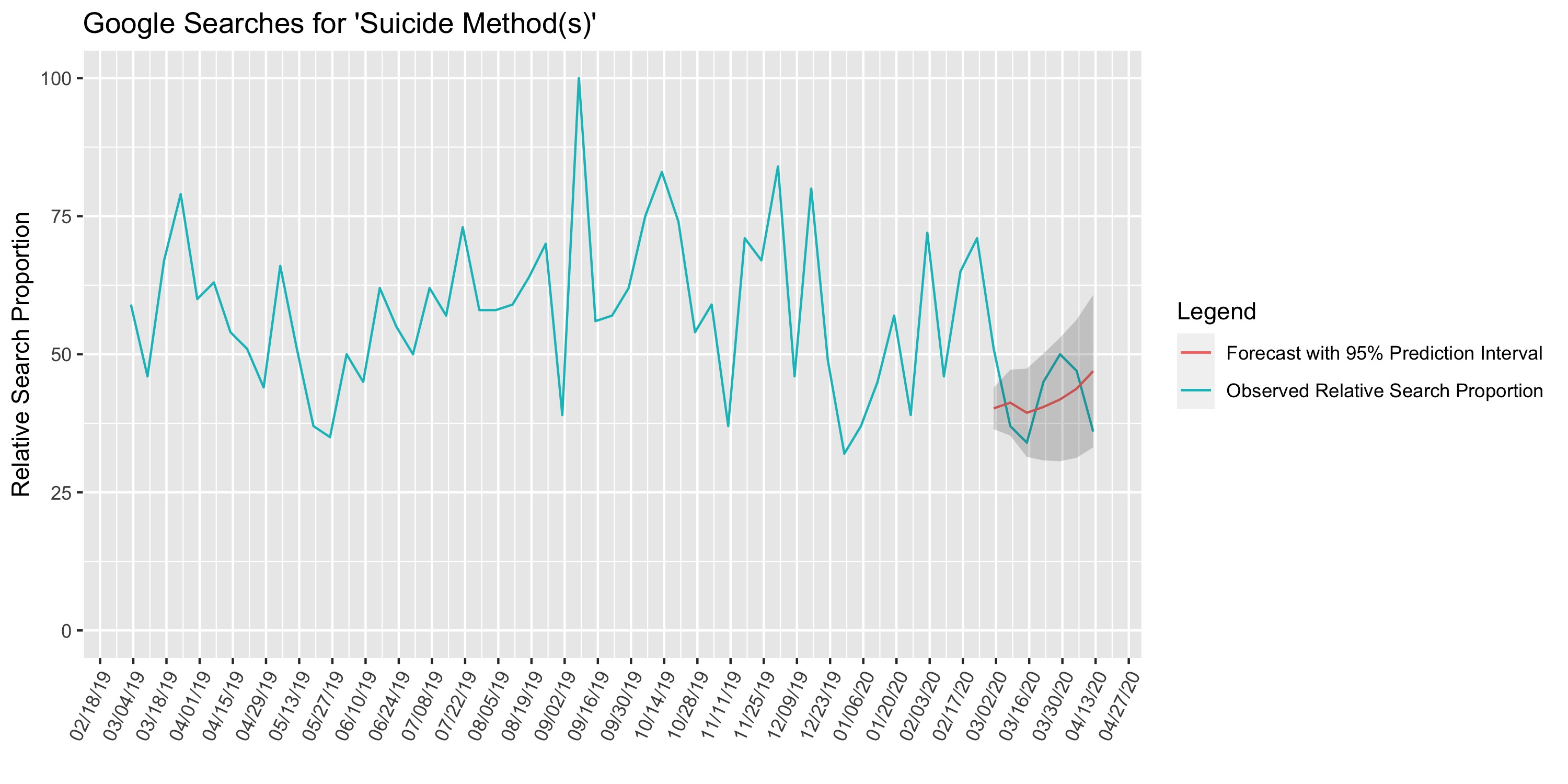


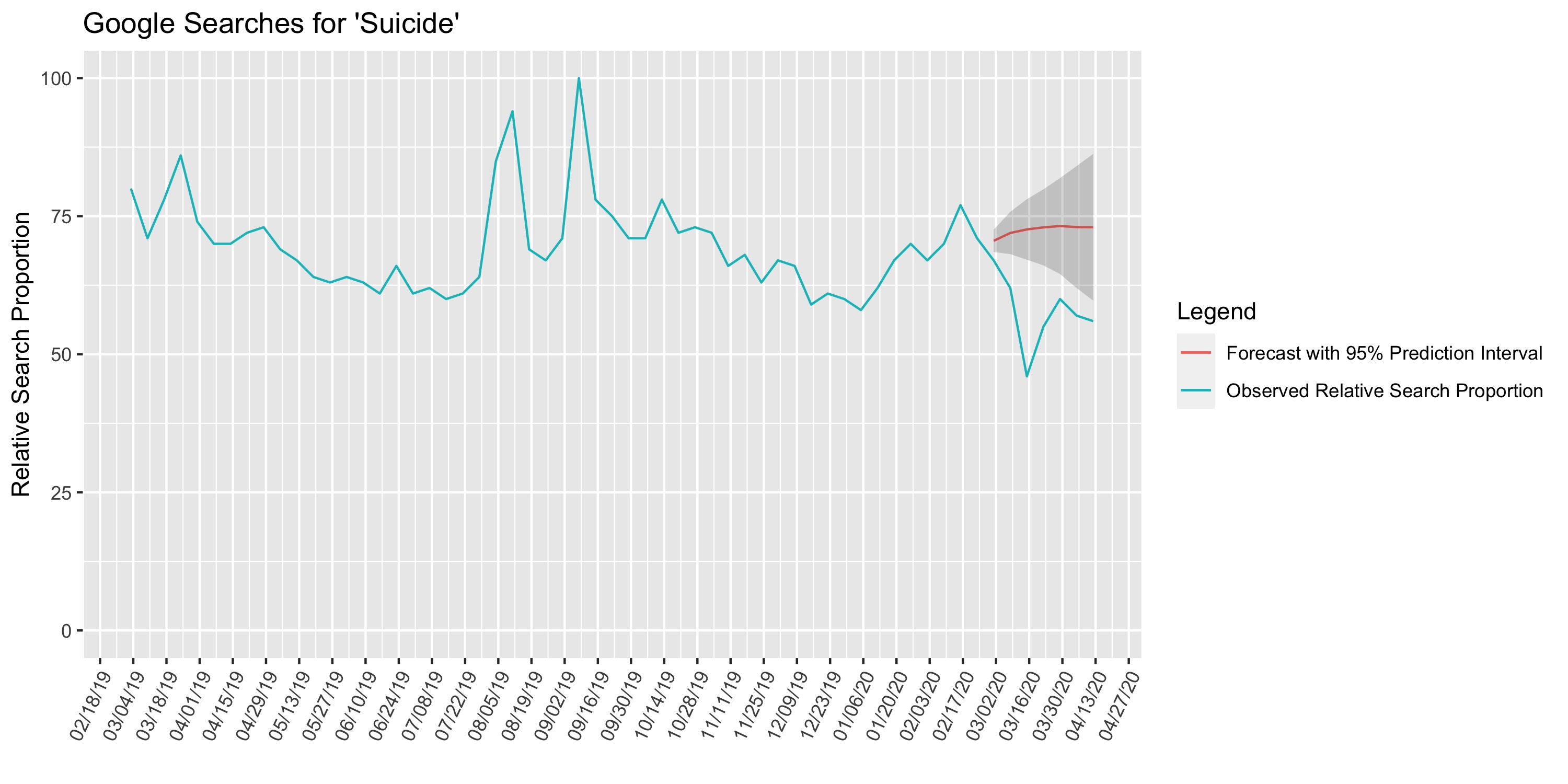

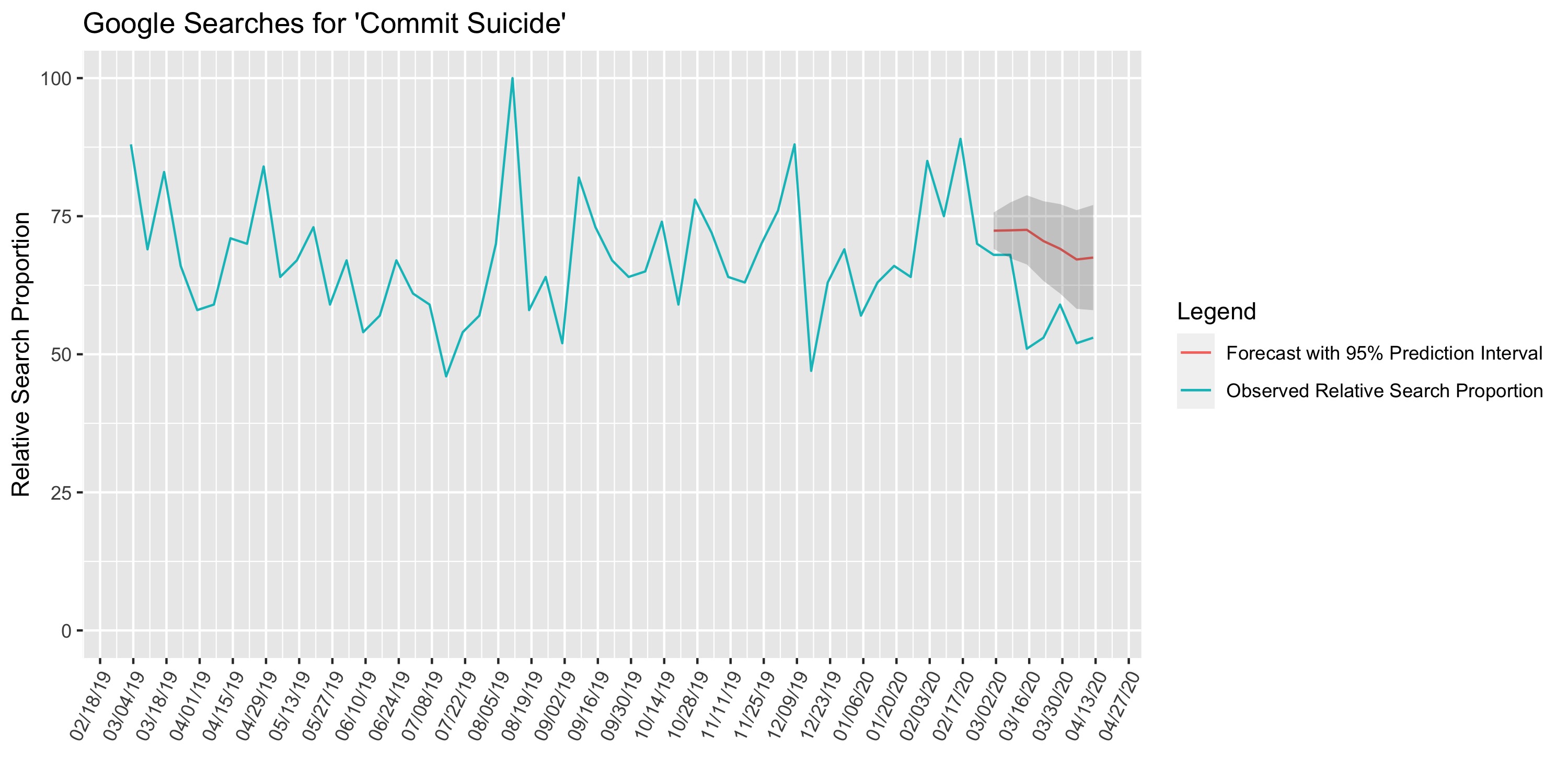

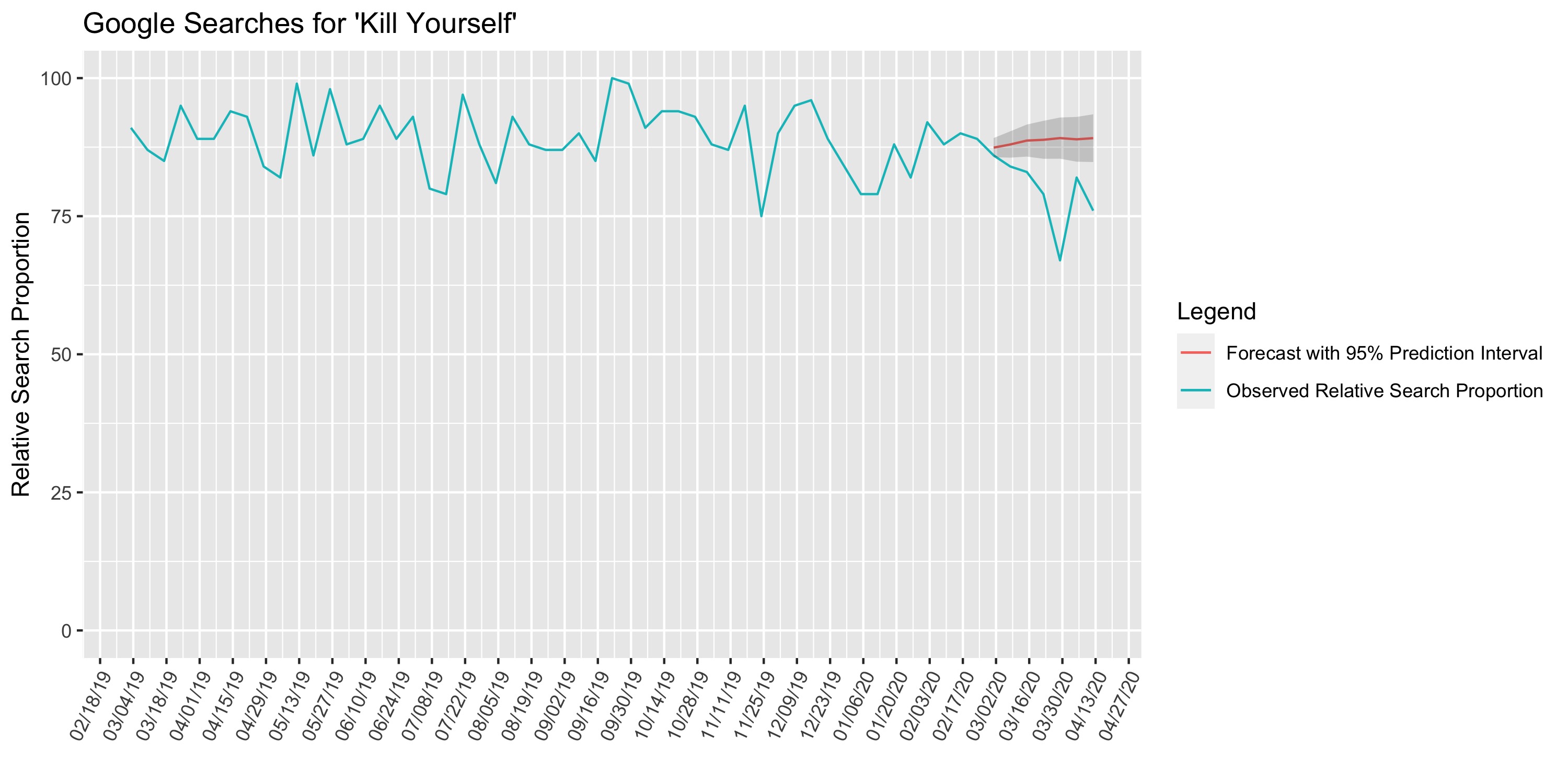


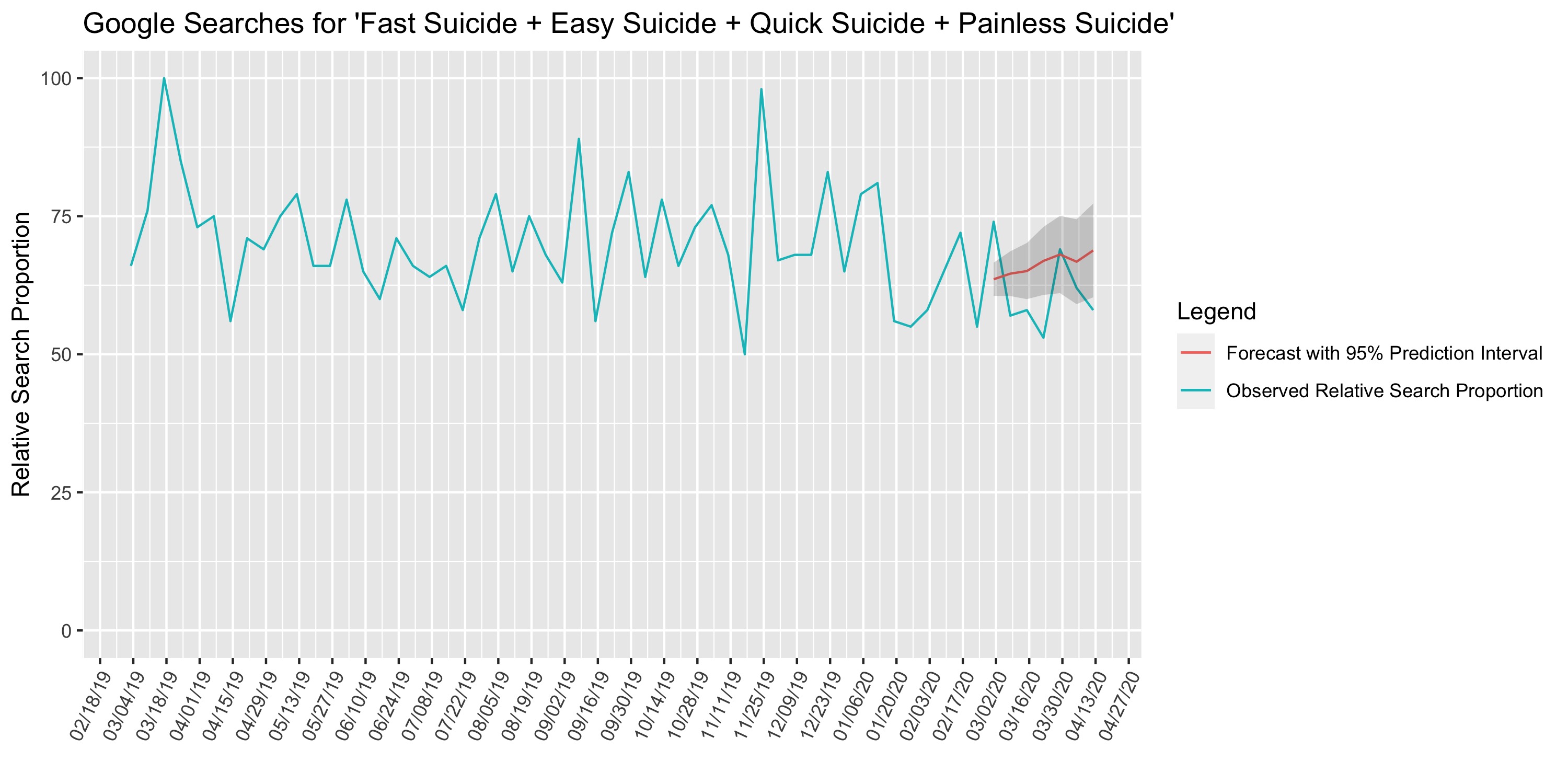


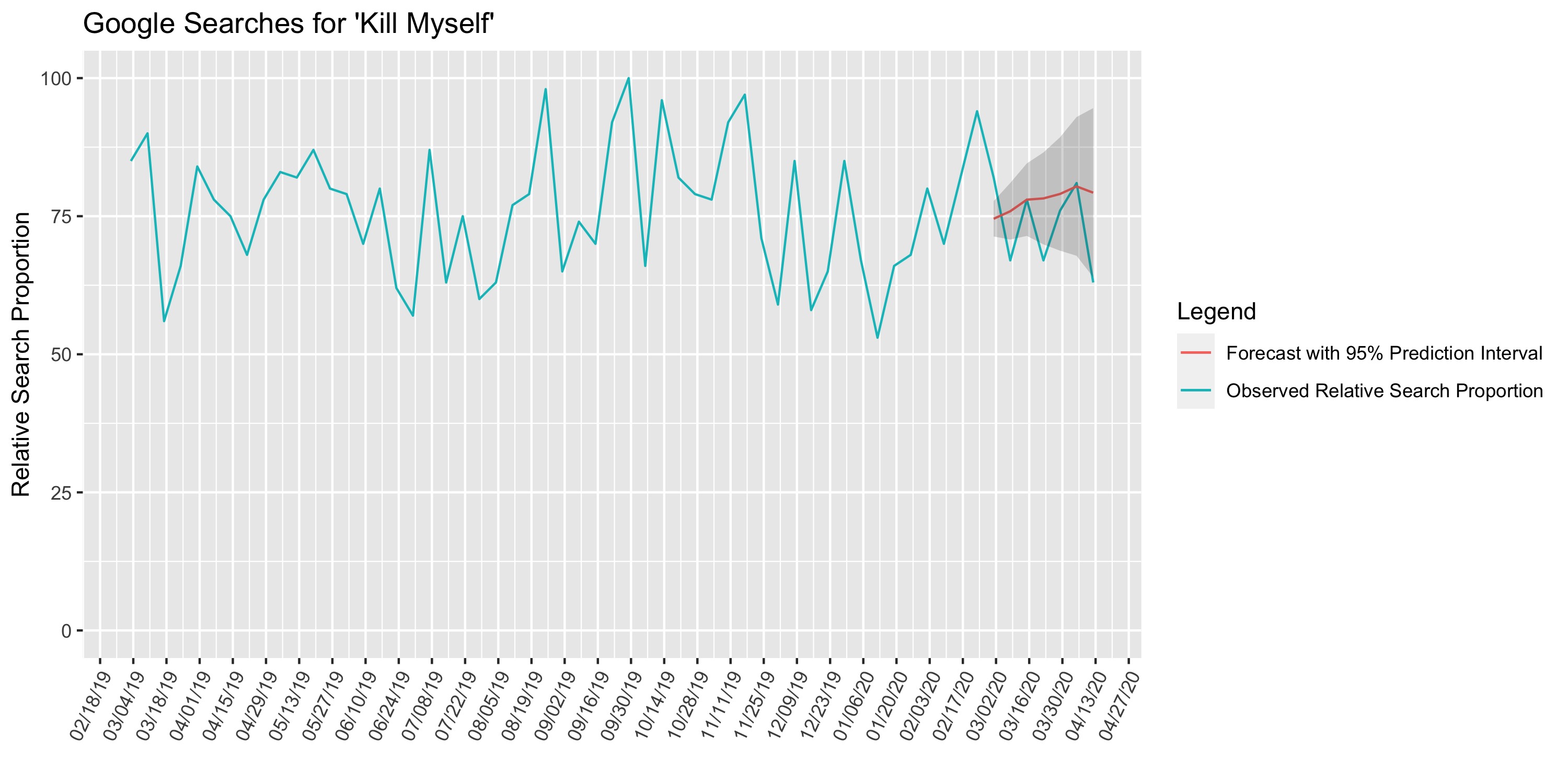


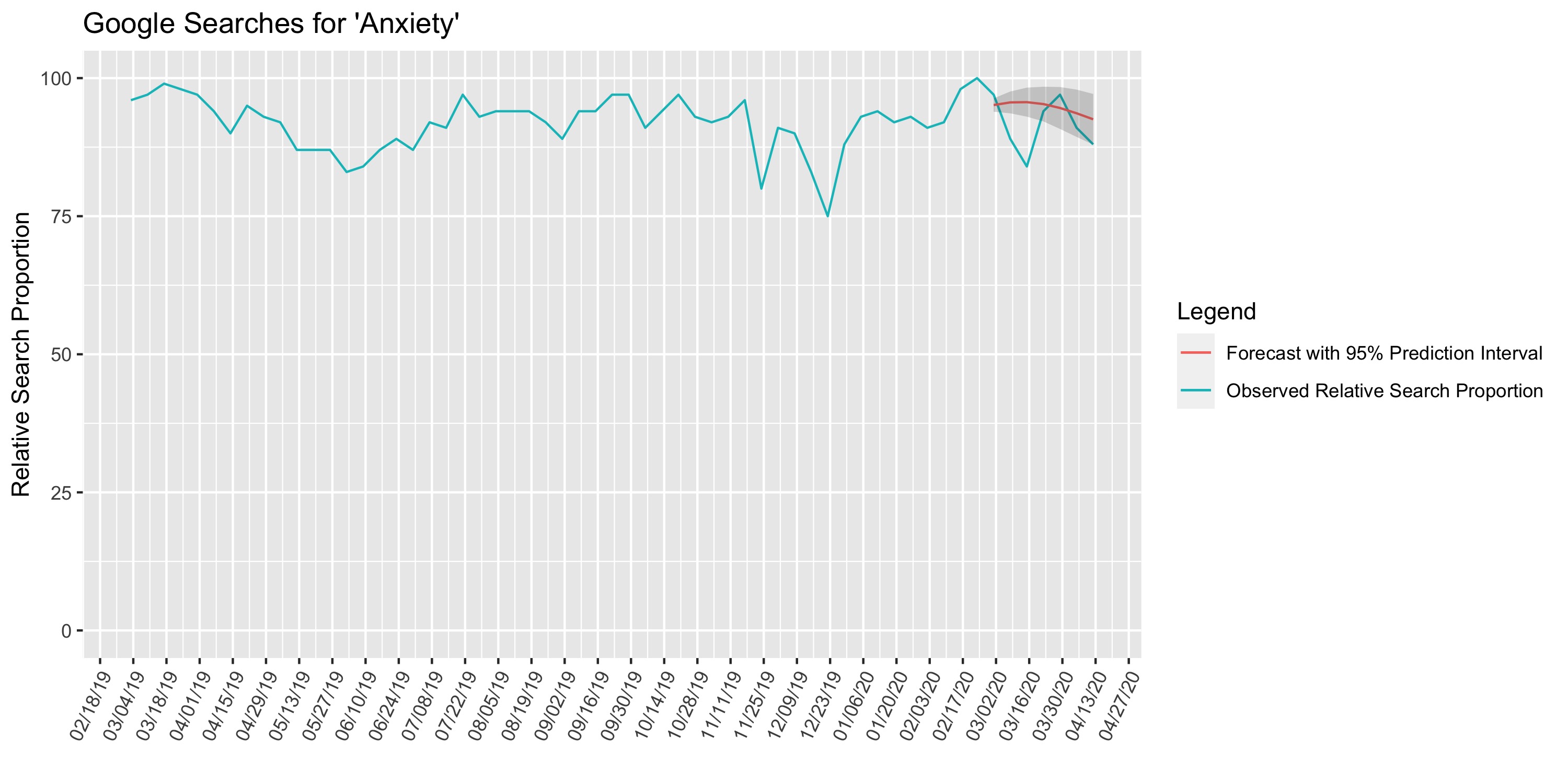


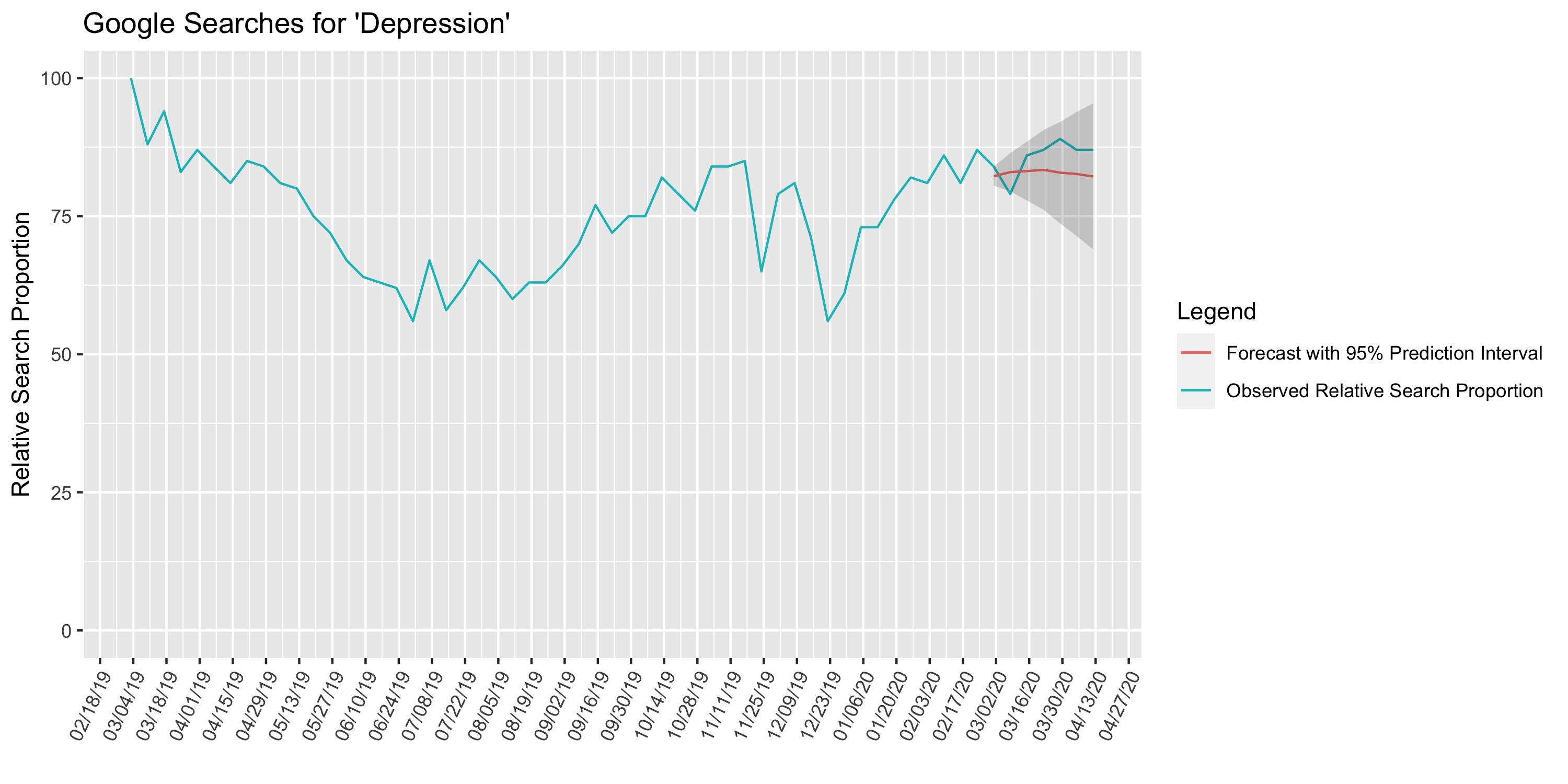


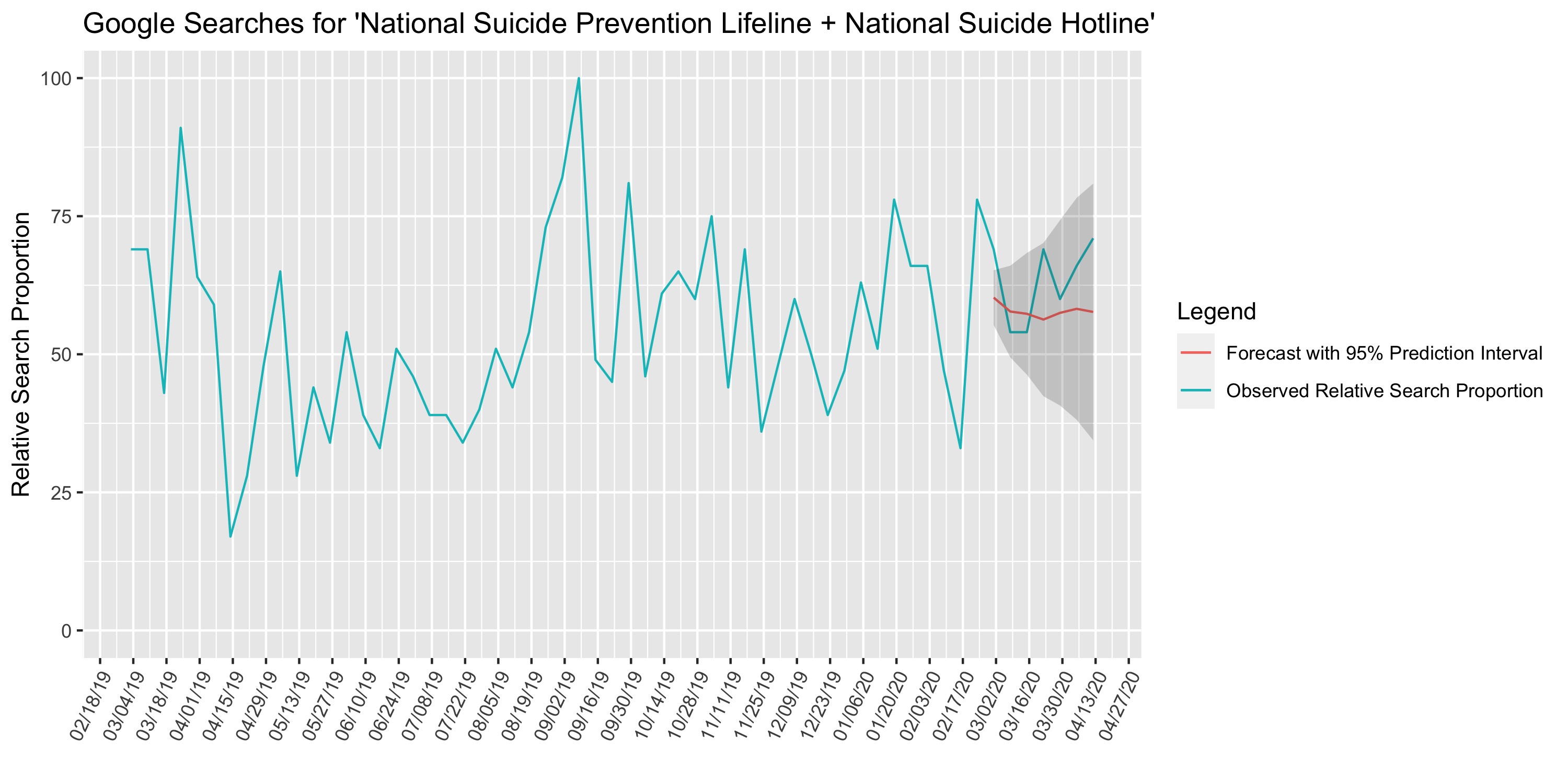


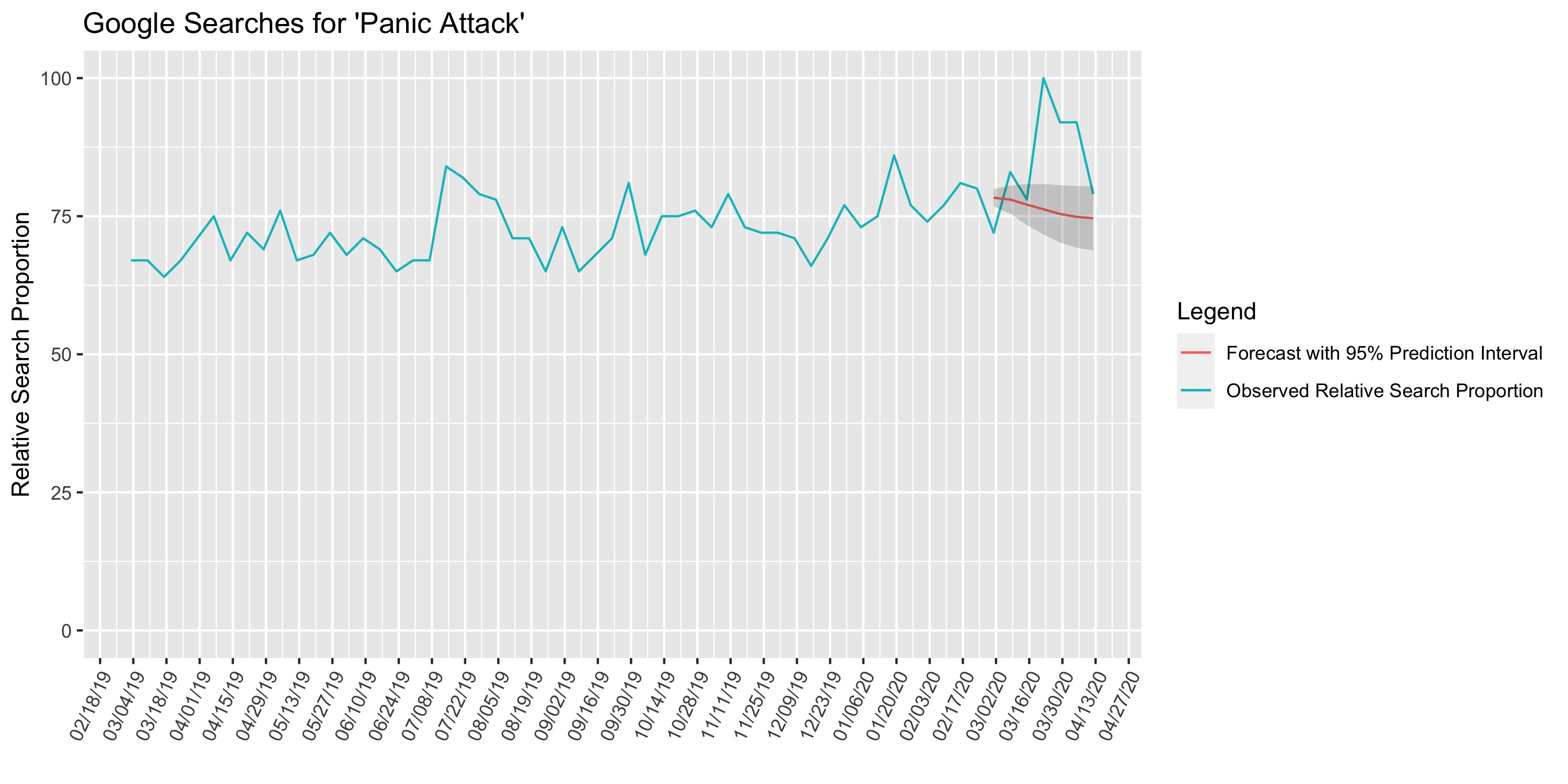


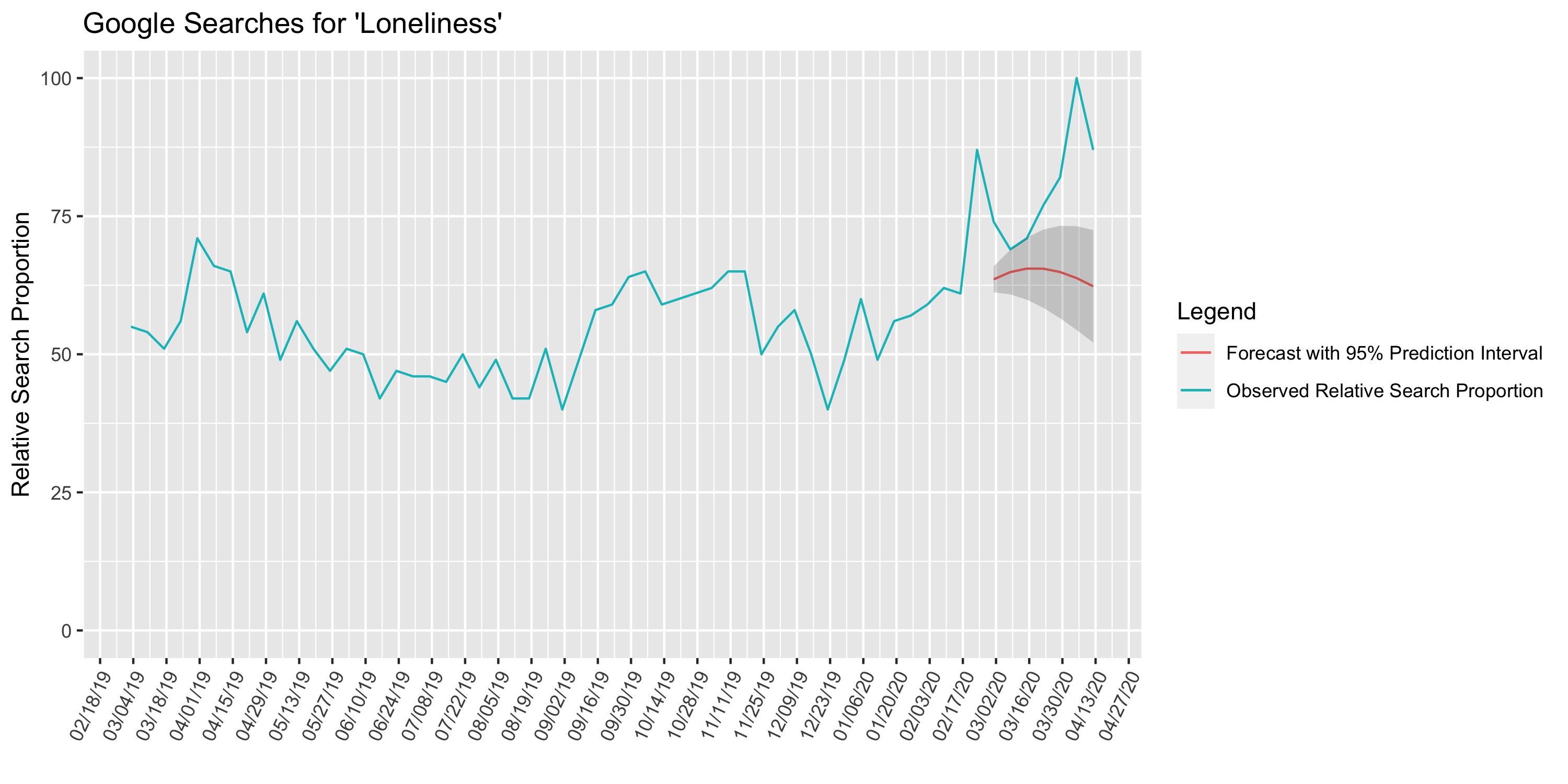


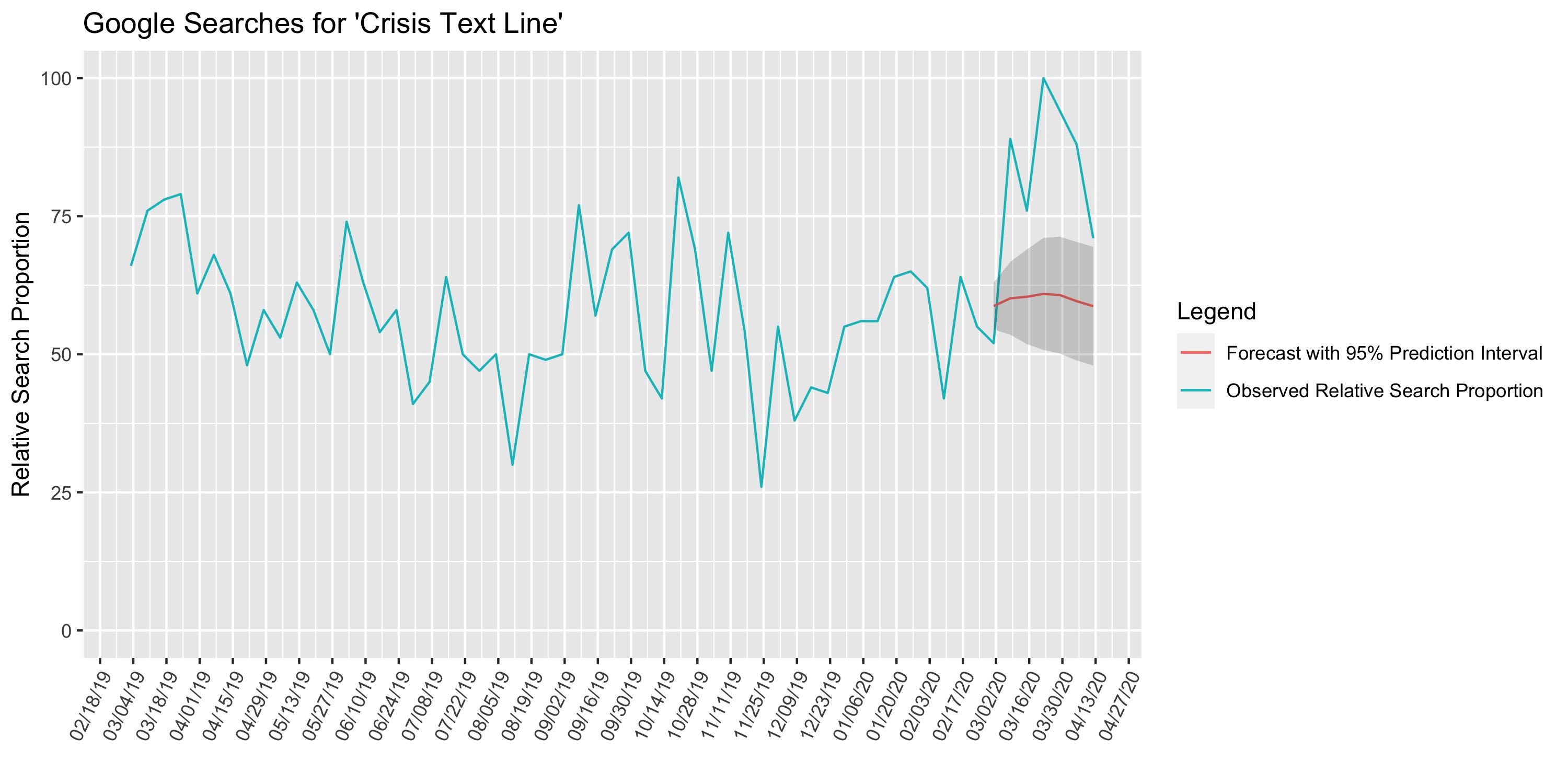


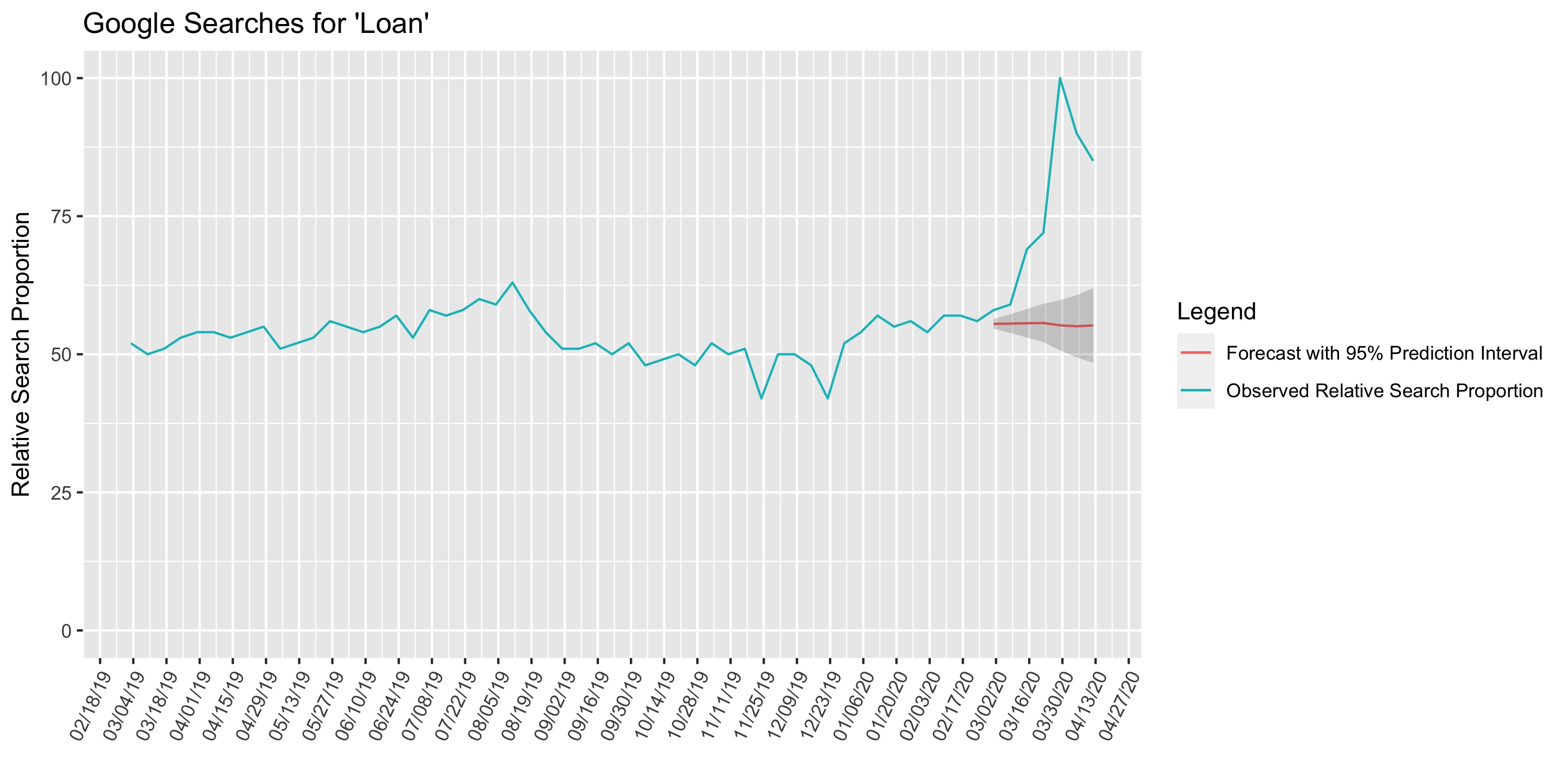


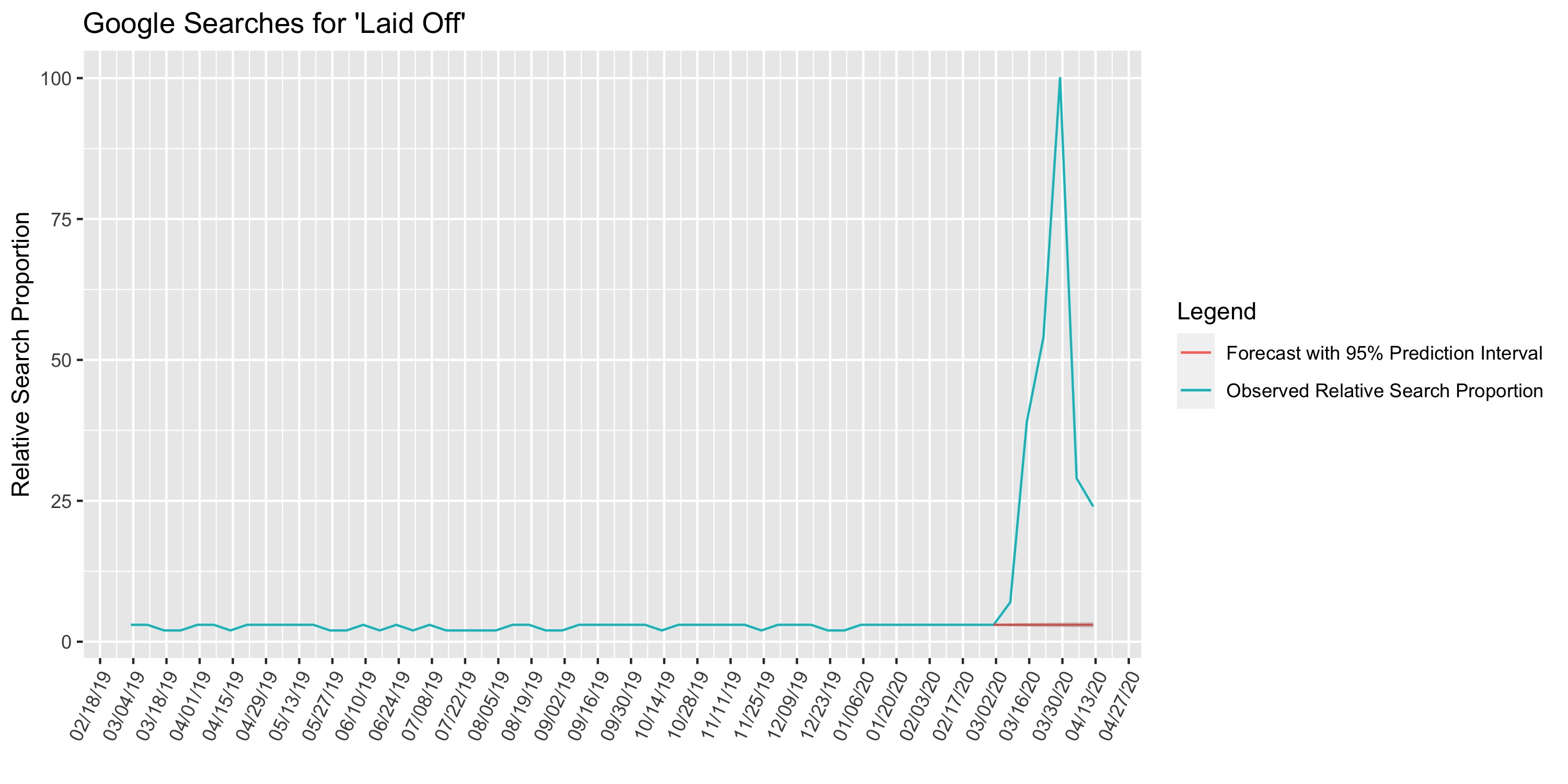


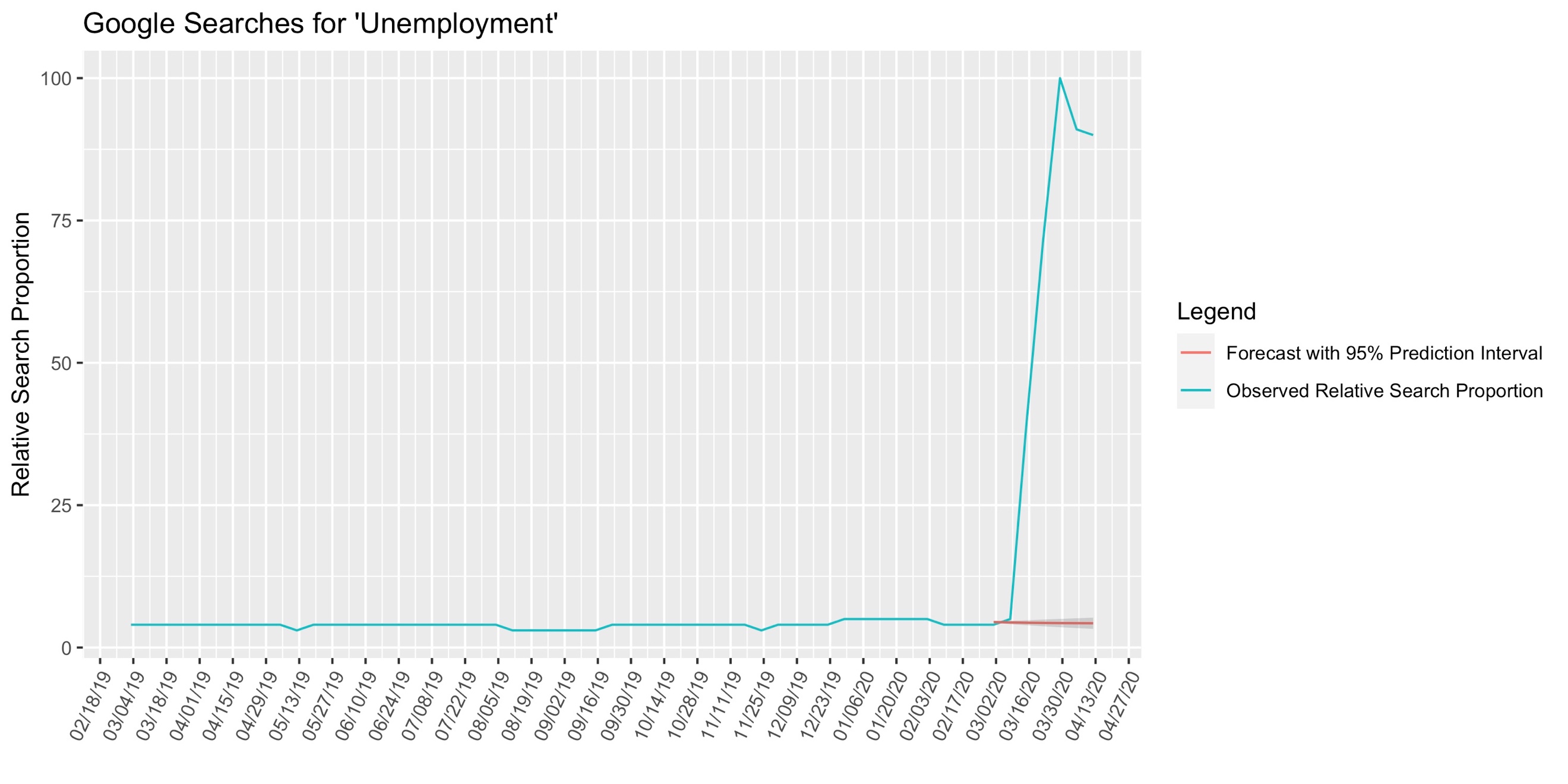


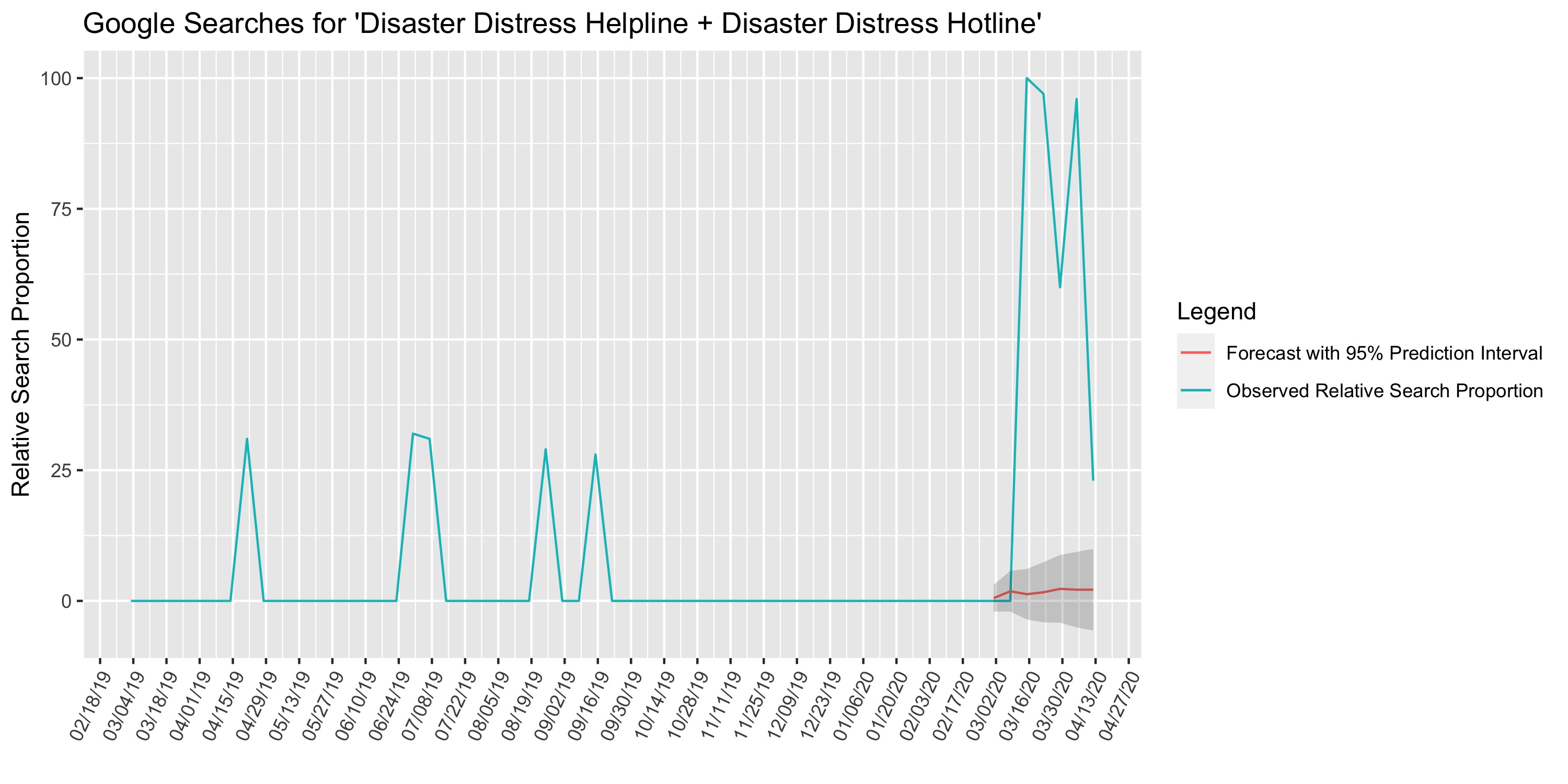


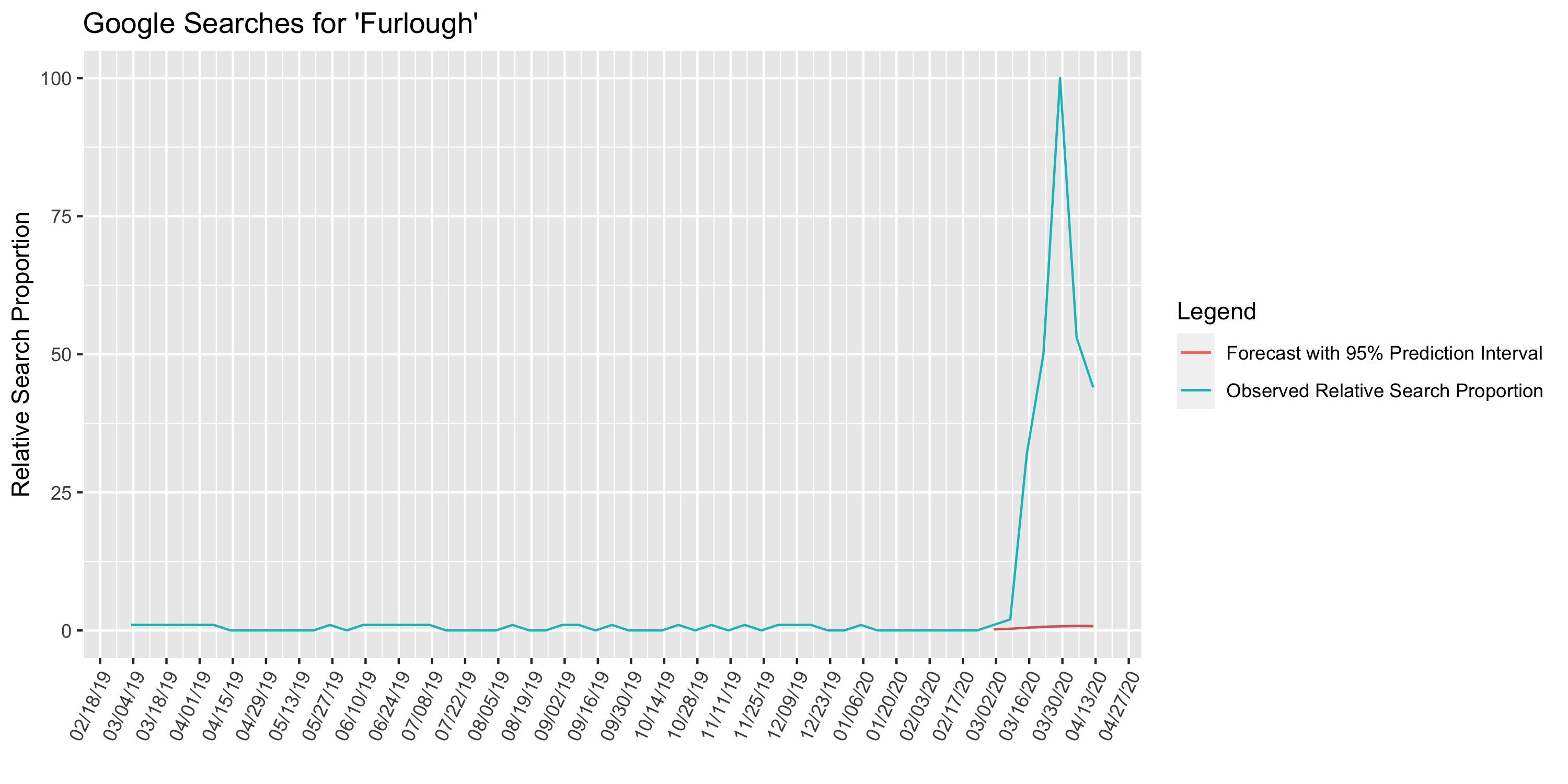

Supplement: S1 File — (DOCX) [file pone.0236777.s001.docx]
